# Association between stunting and early childhood development among children aged 36–59 months in South Asia

**DOI:** 10.1111/mcn.12684

**Published:** 2018-11-29

**Authors:** Yunhee Kang, Víctor M. Aguayo, Rebecca K. Campbell, Keith P. West

**Affiliations:** ^1^ Center for Human Nutrition Johns Hopkins School of Public Health Baltimore Maryland USA; ^2^ Programme Division, UNICEF New York USA

**Keywords:** early childhood development, early childhood education, learning/cognitive development, Multiple Indicator Cluster Surveys (MICS), South Asia, stunting

## Abstract

Stunting (length‐for‐age *z* score < −2) before 2 years of age has shown associations with poor child developmental indicators, but information at the population level is scarce in South Asia, the region with the highest burden of stunting. We examined associations between *z* scores (i.e., height for age [HAZ], weight for age [WAZ], and weight for height [WHZ]) and undernutrition (i.e., stunting [HAZ < −2], wasting [WHZ < −2], and underweight [WAZ < −2]) with learning/cognition and social–emotional development among children 36–59 months of age. Data from Multiple Indicator Cluster Surveys in Bangladesh (*n* = 8,659), Bhutan (*n* = 2,038), Nepal (*n* = 2,253), and Pakistan (Punjab *n* = 11,369 and Sindh *n* = 6,718) were used. Children were considered developmentally “on‐track” in learning/cognition or social–emotional domains if they met specific early child development criteria. Meta‐analysis was conducted to examine regional associations, adjusting for socio‐economic status, early childhood education, and quality of care. In a pooled sample, on‐track learning/cognition development was positively associated with HAZ (*OR* = 1.17, 95% CI [1.07, 1.27]) and WAZ (*OR* = 1.18, 95% CI [1.07, 1.31]) and negatively associated with stunting (*OR* = 0.72, 95% CI [0.60, 0.86]) and underweight (*OR* = 0.75, 95% CI [0.66, 0.86]) but not associated with WHZ or wasting. On‐track development of social–emotional domain was not associated with any *z* scores or undernutrition indicators. Across several countries of South Asia, stunted children were less likely to be developmentally “on track” for learning/cognition. It is likely that interventions that prevent stunting may benefit child development, leading to significant individual and societal gains given the large burden of child stunting in regions like South Asia.

**Key messages**

- In South Asia, stunted and underweight children are at risk of suboptimal learning/cognition development at 3–4 years of age. However, undernutrition may not affect the social–emotional development domain of children aged 3–4 years.
- Interventions that promote linear growth and prevent child stunting may contribute to improve child development outcomes in South Asia.

## 1 INTRODUCTION

Early childhood development is the basis of school readiness, educational achievement, national productivity, and social capital (Daelmans et al., 2017). As a multidimensional and integrated process that links cognitive, motor, and social–emotional self‐regulation skills (Black et al., 2017), brain development is influenced by macronutrient and micronutrient adequacy, inflammation, psychosocial factors (John, Black, & Nelson 3rd, 2017), and, more broadly, the stimulation and stress that covary with socio‐economic well‐being (Walker et al., 2011).

Human neurodevelopmental processes begin within weeks after conception and continue through infancy (Couperus & Nelson, 2006). Animal models, supported by human studies, reveal that gestational nutrient deficiencies may impair neuron proliferation, dendritic branching, and synapse formation and function during pregnancy and infancy (Prado & Dewey, 2014). Postnatally, severe wasting, stunting, and deficiencies in some micronutrients (e.g., iron, zinc, vitamin B12, and iodine) have been shown to be associated with developmental deficits during early to midchildhood (John et al., 2017) and may extend intergenerationally. For example, Jamaican children born to parents who had been stunted before age 2 years scored lower on cognitive development scales than did peers born to parents who were not stunted in early childhood (Walker, Chang, Wright, Osmond, & Grantham‐McGregor, 2015). Trials evaluating prenatal and childhood micronutrient supplementation have revealed positive effects on cognitive and executive functioning performance, educational attainment, learning, and reading ability at a later age compared with controls (Black et al., 2017; Walker et al., 2011).

Nationally representative Demographic and Health Survey and Multiple Indicator Cluster Surveys (MICS) data from 35 low‐ and middle‐income countries (LMICs) estimate that approximately 81.0 million (33.0%) children aged 3 and 4 years have low cognitive and/or social–emotional development (McCoy et al., 2016). In an analysis of cross‐sectional data from 11 LMICs, each unit increase in height‐for‐age *z* score was associated with a 0.24 *SD* increase in cognitive score before age 2 years, although the association was attenuated to 0.09 *SD* from 2 to 12 years of age (Sudfeld et al., 2015). Globally, the largest number of stunted children resides in South Asia, where ~33.3%, or 58.7 million, of children under 5 years of age are affected (UNICEF, WHO, & World Bank Group, 2018). Furthermore, the region also harbours the second‐highest number (27.8 million) of children with low cognitive and socioemotional Early Childhood Development Index (ECDI) test scores after the Sub‐Saharan Africa region (McCoy et al., 2016), justifying our interest to explore associations between preschool child nutritional status and development in South Asia.

Accessing population‐level data from recent‐year MICS in Bangladesh, Bhutan, Nepal, and Pakistan, the present study examined associations between undernutrition and learning/cognition and social–emotional development indicators among South Asian children aged 36 to 59 months. We further tested the consistency of effect estimates across the region by meta‐analysis.

## 2 PARTICIPANTS AND METHODS

### 2.1 Data sources

Child development indicators were introduced in MICS‐Round 4 (http://mics.unicef.org/) to collect valid and reliable data on child developmental milestones at the population level. For this study, we used the most recent round of MICS in South Asian countries as the primary source of data. The survey datasets included in our analysis were Bangladesh (Bangladesh Bureau of Statistics & UNICEF Bangladesh, 2014); Bhutan (MICS‐Round 4; National Statistics Bureau, 2011); Nepal (Nepal Central Bureau of Statistics, 2015); Punjab province, Pakistan (Bureau of Statistics Punjab & UNICEF Punjab., 2016); and Sindh province, Pakistan (Sindh Bureau of Statistics & UNICEF, 2015). Afghanistan's MICS‐Round 5 was not included in the analysis due to the absence of child anthropometric measurements in the dataset. All datasets were publicly available prior to March 15, 2017.

### 2.2 Available variables

An ECDI assessment, introduced into the fourth and fifth rounds of MICS, was designed to assess development in children aged 36–59 months (UNICEF, 2014). The ECDI is based on responses to 10 yes/no questions by primary caregivers. An initial validation study for ECD was conducted in Jordan and the Philippines, comprising 900 children aged 3–4 years in each country, with reliability testing for a subsample (Loizillon, Petrowski, Britto, & Cappa, 2017). After a series of factor analyses, ECD was constructed as long (48‐item) and short (18‐item) sets of items covering six child development domains (Janus & Duku, 2012). The 18‐item set draft ECDI was field tested in Kenya (Janus & Duku, 2011; Plowman, 2014), and the final revisions were made, leading to the current 10‐item set for four child development domains: literacy‐numeracy (three items), physical (two items), learning/cognition (two items), and social–emotional (three items). Recently, McCoy et al. (2016) demonstrated that ECDI items designed to assess literacy/numeracy were too advanced for 3‐ and 4‐year‐old children and that the sensitivity of the physical domain module was low, detecting only more extreme conditions. For these reasons, our current analysis was restricted to the learning/cognition and socioemotional domains.

Based on ECDI criteria (UNICEF, 2014), learning/cognition development is considered to be on track “if a child can follow simple directions on how to do something correctly and/or when given something to do, is able to do it independently” (UNICEF, 2014). The social–emotional domain is on track “if child can do at least two of the following: gets along well with other children; does not kick, bite or hit other children; and/or does not get distracted easily.”

#### 2.2.1 Undernutrition variables

The prevalence of undernutrition (i.e., stunting, underweight, and wasting) and nutrition status *z* scores (height for age [HAZ], weight for age [WAZ], and weight for height [WHZ]) derived from anthropometric measurements during the MICS were included as independent variables. The prevalence of stunting, underweight, and wasting was defined as the percent of children whose HAZ, WAZ, and WHZ, respectively, were two standard deviations below the WHO reference median for children of the same month of age and sex (WHO Multicentre Growth Reference Study Group, 2006).

#### 2.2.2 Confounding variables

We selected socio‐economic, early childhood education, and quality of care variables as potential confounders to see how they affected the associations between undernutrition and child development. Socio‐economic variables were selected at the individual, maternal, and household levels if they were conceptually related to child nutritional status and development and were available in the five surveys. Household variables included rural/urban residence, household wealth index, household size (greater than five members or not), gender of household head, use of improved drinking water sources, and use of improved toilet facilities. Household wealth index quintiles were generated by principal components analysis using information on the dwelling characteristics, including water sources and toilet facilities, fuel types for cooking, durable assets, livestock, electricity, and other characteristics related to household wealth. Maternal education attainment was also included, as were child sex and age. Reported episodes of diarrhoea and cough in the past 2 weeks were also included as individual level characteristics.

Early childhood education and quality of care were assessed in the child development section of the MICS‐Rounds 4 and 5 (UNICEF, 2013), based on the mother's or caretaker's report, as they are considered to be essential to child development. Attendance at early childhood education was defined as attending an early childhood education programme (i.e., private or government facility including kindergarten or community child care). Support for learning was defined as any family member over 15 years of age having engaged in four or more activities to promote learning and school readiness in the 3 days prior to the interview: (a) reading a book(s) or looking at a picture book(s) with the child; (b) telling a story to the child; (c) singing song(s), including lullabies, to or with their child; (d) taking the child outside the home, compound, yard, or enclosure; (e) playing with the child; and (f) naming, counting, or drawing things with the child. Availability of children's books was defined as having three or more children's books in the home. Availability of playthings was defined as the child playing with two or more types of playthings out of homemade toys, toys from a shop, and household/outside objects (sticks, rocks, animal shells, or leaves). Inadequate care was defined as the child's being left alone or in the care of another child younger than 10 years for more than 1 hr at least once in the last week.

### 2.3 Statistical analysis

The two‐stage cluster sampling design of the original surveys, which randomly selected households with children under 5 years in enumeration areas (primary sampling unit) from the national census, was taken into account in all analyses. “svy” commands were used to estimate summary statistics and associations at the population level. Learning/cognition and social–emotional variables were dichotomized as being on track versus not. To see the associations between *z* scores/undernutrition and developmental indicators, we first explored the bivariate logistic regression analysis (Model 1). Next, the models were adjusted for socio‐economic variables (Model 2), and finally models were adjusted for early childhood education and quality of care in addition to the socio‐economic variables (Model 3). We conducted meta‐analysis and created forest plots. Adjusted odds ratios and 95% CIs from Model 2 and Model 3 were log transformed, and the “metan” command with the option of “eform effect (OR)” was used. Heterogeneity (*I* ^2^) of association across pooled country datasets was measured; *I* ^2^ greater than 50% or *I* ^2^ *P* value < 0.05 indicated significant heterogeneity (Higgins, Thompson, Deeks, & Altman, 2003). Heterogeneity was incorporated into a random‐effects model that allows different population estimates of effect size to be combined as recommended by the Cochrane guidelines (Higgins et al., 2003). Stata Version 14 (StataCorp LP, College Station, TX) was used for all statistical analyses.

### 2.4 Ethical considerations

This secondary data analysis was deemed exempt from ethics review by the Institutional Review Board, Johns Hopkins School of Public Health, as no human subjects work was conducted as part of this project.

## 3 RESULTS

### 3.1 General characteristics

This study included nationally representative children 36 to 59 months of age in Bangladesh (*n* = 8,659), Bhutan (*n* = 2,038), Nepal (*n* = 2,253), and Punjab (*n* = 11,369) and Sindh (*n* = 6,718) provinces in Pakistan (Table 1). The proportion of households from rural areas varied from 53.7% in Sindh province in Pakistan to 86.7% in Nepal. The proportion of households with five or more members was greatest in Sindh, Pakistan (92.3%), and smallest in Bangladesh (54.0%). The proportion of female‐headed households was highest in Nepal (27.4%), followed by Bhutan (22.8%), and low in both provinces in Pakistan (4.4% and 5.8% in Sindh and Punjab, respectively). In all countries, most households had access to improved drinking water (89.5–96.4%), and roughly half of households used improved toilet facilities (50.0–60.0%). The proportion of mothers who did not complete primary education or had no formal education was lowest in Bangladesh (40.7%) and highest in Bhutan (67.1%). The proportion of children experiencing diarrhoea and cough in the past 2 weeks varied from 1.8% in Bangladesh to 18.3% in Sindh, Pakistan, and 7.7% in Punjab, Pakistan, to 45.4% in Bhutan, respectively.

**Table 1:** Selected household‐, maternal‐, and child‐level characteristics among children 36 to 59 month of age in Bangladesh, Bhutan, Nepal, and Pakistan, MICS 4 or MICS 5 (2010–2014)

Characteristics	Bangladesh (*n* = 8,659)	Bhutan (*n* = 2,038)	Nepal (*n* = 2,253)	Punjab, Pakistan (*n* = 11,369)	Sindh, Pakistan (*n* = 6,718)
	%	%	%	%	%
*Household level*					
Resident area					
Urban	20.0	30.8	13.3	31.3	46.3
Rural	80.0	69.2	86.7	69.9	53.7
Household size ≥ 5	54.0	71.7	68.8	90.0	92.3
Female household head	9.9	22.8	27.4	5.8	4.4
Use of improved drinking water source	89.5	96.4	93.9	94.8	90.4
Use of improved toilet facility	50.0	50.3	52.2	60.0	56.6
*Maternal level*					
Education					
None/primary incomplete	40.7	67.1	48.7	50.9	58.8
Primary	15.4	13.4	16.8	27.0	19.0
Secondary	32.1	19.5	19.2	12.1	10.9
Higher	11.7	—a	15.2	10.0	6.9
*Child level*					
Age					
36–47 months	50.3	50.5	51.0	51.7	51.5
48–59 months	49.7	49.5	49.0	48.3	48.5
Female sex	48.1	48.7	49.5	49.5	48.2
Having diarrhoea^b^	1.8	17.3	9.2	10.4	18.3
Having cough	22.0	45.4	23.0	7.7	25.8
*Early childhood education and quality of care*					
Attendance to early childhood education^c^	13.5	10.3	51.3	26.1	18.0
Support for learning^d^	87.4	72.5	78.1	51.6	56.2
Availability of children's books^e^	17.9	12.6	9.7	15.3	11.4
Availability of playthings^f^	74.3	62.2	72.9	61.1	73.2
Inadequate care^g^	14.4	18.4	26.0	7.9	18.7

*Note*. MICS: Multiple Indicator Cluster Surveys.

a Bhutan MICS has only three categories of maternal education: none, primary, and secondary.

b Having an episode of diarrhoea (or cough) during the 2 weeks preceding the survey, based on mother's (caregiver's) report.

c % of children who are attending an early childhood education programme.

d During the 3 days prior to the interview, any family member over 15 years of age had been engaged in four or more of the following activities with the child: (a) reading a book(s) or looking at a picture book(s) with the child; (b) telling a story to the child; (c) singing song(s), including lullabies, to or with the child; (d) taking the child outside the home, compound, yard, or enclosure; (e) playing with the child; and (f) naming, counting, or drawing things with the child.

e % of children who have three or more children's books.

f % of children who play with two or more types of playthings.

g % of children who were left alone or in the care of another child younger than 10 years for more than 1 hr at least once in the last week.

The proportion of children attending early childhood education programmes was low overall, ranging from 10.3% in Bhutan to 51.3% in Nepal (Table 1). The proportion reporting support for child learning from family members was lowest in Pakistan (51.6% in Punjab and 56.2% in Sindh) and highest in Bangladesh (87.4%). Availability of three or more children's books was low at <20% in all countries, whereas the availability of playthings was moderately high at 61.1–74.3%. The proportion of children with inadequate care was largely low, ranging from 7.9% in Punjab, Pakistan, to 26.0% in Nepal.

### 3.2 Nutritional status

**Figure 1:**
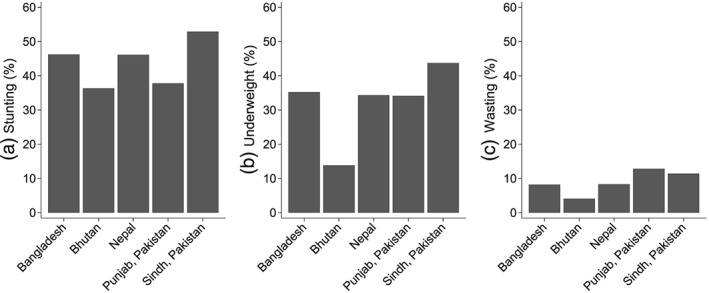
Prevalence of undernutrition among children 36–59 months of age in Bangladesh, Bhutan, Nepal, and Pakistan, Multiple Indicator Cluster Surveys (MICS) 4 or MICS 5 (2010–2014). (a) Stunting, (b) underweight, and (c) wasting

The prevalence of stunting varied: 36.3% in Bhutan, 37.8% in Punjab, Pakistan, 46.1% in Nepal, 46.2% in Bangladesh, and 52.9% in Sindh, Pakistan (Figure 1). Underweight was also notably high in Sindh, Pakistan (43.7%). The prevalence of underweight was comparable in Bangladesh, Nepal, and Punjab, Pakistan (34–35%). Bhutan had the lowest prevalence of underweight (13.8%). The prevalence of wasting was moderately high, ranging from 8.2% in Bangladesh to 12.8% in Sindh, Pakistan, except for Bhutan, where the prevalence was 4.1%.

Suboptimal linear growth and ponderal growth were common in the region. The mean HAZ ranged from −1.62 *Z* in Bhutan to −2.16 *Z* in Sindh, Pakistan. The mean WAZ ranged from −1.02 *Z* in Bhutan to −1.84 *Z* in Sindh, Pakistan. The mean WHZ of Bhutanese children was close to 0, whereas other countries in the region presented faltering in WHZ with the worst mean *z* score of −0.90 in Punjab, Pakistan (Table S1).

### 3.3 Early childhood development indicators

**Figure 2:**
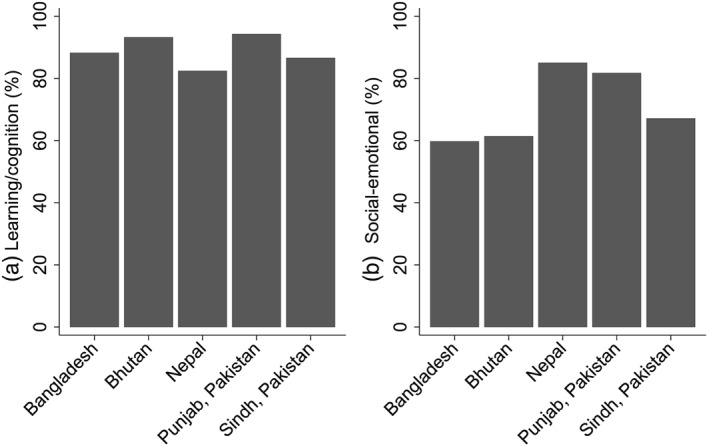
Prevalence of being on track for learning/cognition and social–emotional development indicators among children 36–59 months of age in Bangladesh, Bhutan, Nepal, and Pakistan, Multiple Indicator Cluster Surveys (MICS) 4 or MICS 5 (2010–2014). (a) Learning/cognition; (b) social–emotional

The proportion of children who had on‐track development in the learning domain was high, ranging from 82.4% in Nepal to 93.2% in Bhutan and 94.3% in Punjab, Pakistan (Figure 2). The proportion of children who had on‐track development in the social–emotional domain ranged from 59.7% in Bangladesh, to 85.0% in Nepal.

### 3.4 Association between nutritional status and learning/cognitive and socioemotional development

#### 3.4.1 Learning/cognitive development

Stunting was associated with lower odds of learning development in the pooled samples (*OR* = 0.72, 95% CI [0.60, 0.86], in Model 3); similarly, underweight was associated with poorer learning outcomes (*OR* = 0.75, 95% CI [0.66, 0.86]) in the pooled samples (Table 2). However, wasting was not associated with learning developmental domain (*OR* = 0.98, 95% CI [0.70, 1.37]) in the pooled samples. A significant heterogeneity with stunting (*I* ^2^ = 61.1%; *P* = 0.04) and wasting (*I* ^2^ = 74.1%; *P* = 0.004) in the pooled samples was found, but the pooled *OR* was accepted by using random‐effects meta‐analysis. A consistent direction of association between stunting and learning domain was found across five datasets. When additionally adjusted for early childhood education and quality of care, the magnitude of associations with stunting, underweight, and wasting remained equal to models that adjusted only socio‐economic variables (*OR* = 0.69 to *OR* = 0.72 in stunting; *OR* = 0.73 to *OR* = 0.75 in underweight; *OR* = 0.97 to *OR* = 0.98 in wasting).

**Table 2:** Odds of being on track for learning development among undernourished children relative to children of normal nutritional status, 36–59 months of age, in Bangladesh, Bhutan, Nepal, and Pakistan^a^

Variables	Model 1 (crude)^b^			Model 2^c^			Model 3^d^		
	*OR* e	95% CI	*P*	*OR*	95% CI	*P*	*OR*	95% CI	*P*
Stunting									
Bangladesh	0.51	[0.42, 0.61]	<0.001	0.55	[0.45, 0.68]	<0.001	0.56	[0.45, 0.69]	<0.001
Bhutan	0.71	[0.47, 1.07]	0.10	0.72	[0.49, 1.07]	0.10	0.73	[0.50, 1.06]	0.10
Nepal	0.79	[0.58, 1.06]	0.12	0.92	[0.67, 1.26]	0.60	1.07	[0.76, 1.51]	0.69
Punjab, Pakistan	0.55	[0.46, 0.66]	<0.001	0.69	[0.57, 0.84]	<0.001	0.69	[0.57, 0.84]	<0.001
Sindh, Pakistan	0.46	[0.37, 0.58]	<0.001	0.69	[0.55, 0.86]	<0.001	0.72	[0.58, 0.90]	<0.001
All countries^f^ *I* ^2^				46.3%	*P* = 0.11		61.1%	*P* = 0.04	
All countries				0.69	[0.59, 0.80]	<0.001	0.72	[0.60, 0.86]	<0.001
Underweight									
Bangladesh	0.72	[0.59, 0.88]	<0.001	0.73	[0.59, 0.90]	<0.001	0.74	[0.60, 0.91]	0.01
Bhutan	0.70	[0.40, 1.21]	0.20	0.68	[0.39, 1.19]	0.18	0.66	[0.38, 1.16]	0.15
Nepal	0.76	[0.55, 1.06]	0.10	0.90	[0.63, 1.28]	0.57	1.06	[0.73, 1.54]	0.76
Punjab, Pakistan	0.65	[0.55, 0.77]	<0.001	0.78	[0.66, 0.94]	0.01	0.78	[0.66, 0.94]	0.01
Sindh, Pakistan	0.46	[0.39, 0.56]	<0.001	0.65	[0.54, 0.78]	<0.001	0.66	[0.55, 0.79]	<0.00
All countries *I* ^2^				0.0%	*P* = 0.46		28.8%	*P* = 0.23	
All countries				0.73	[0.66, 0.81]	<0.001	0.75	[0.66, 0.86]	<0.001
Wasting									
Bangladesh	1.27	[0.92, 1.75]	0.15	1.27	[0.91, 1.78]	0.16	1.27	[0.90, 1.79]	0.17
Bhutan	1.60	[0.32, 7.85]	0.56	1.89	[0.41, 8.72]	0.42	1.93	[0.39, 9.48]	0.42
Nepal	1.09	[0.66, 1.79]	0.73	1.47	[0.88, 2.47]	0.14	1.52	[0.90, 2.58]	0.12
Punjab, Pakistan	0.63	[0.50, 0.79]	<0.001	0.68	[0.53, 0.86]	<0.001	0.67	[0.53, 0.86]	<0.001
Sindh, Pakistan	0.69	[0.53, 0.89]	<0.001	0.76	[0.59, 0.98]	0.03	0.76	[0.59, 0.97]	0.03
All countries *I* ^2^				73.1%	*P* = 0.005		74.1%	*P* = 0.004	
				0.97	[0.70, 1.35]	0.88	0.98	[0.70, 1.37]	0.88

a Learning/cognition development is considered to be on track if child can follow simple directions on how to do something correctly and/or when given something to do, and is able to do it independently.

b Model 1: Crude bivariate model.

c Model 2: Adjusted for location (rural vs. urban), household wealth quintile, household size, type of household head (male vs. female), improved drinking water source (yes/no), improved toilet facility (yes/no), maternal education, child age and sex, episodes of diarrhoea and cough in the past 2 weeks (yes/no), and study design effect.

d Model 3: In addition to all confounding variables used in Model 2, attendance to early childhood education, support for learning, availability of children's books, availability of playthings, and inadequate care were adjusted for in Model 3.

e For all odds ratio estimates, the reference groups are children who are not stunted (HAZ ≥ −2), underweight (WAZ ≥ −2), or wasted (WHZ ≥ −2).

f *I* ^2^ is defined as the percentage of total variation across studies that is due to heterogeneity.

In Bangladesh, stunting and underweight predicted lower odds of achieving learning development. In Bhutan, only severely stunted children (height‐for‐age *z* score < −3) showed marginally lower odds of achieving learning development (data not shown). No nutritional indicators were associated with learning development in Nepal. In Pakistan, stunting, underweight, and wasting predicted lower odds of achieving on‐track learning development.

When testing with continuous nutritional status *z* scores, we found high heterogeneity in the effect size across samples, but the pooled *OR* was accepted by using random‐effects meta‐analysis (Table 3). In the pooled samples, HAZ and WAZ were associated with on‐track development of learning/cognition (*OR* = 1.17, 95% CI [1.07, 1.27], and *OR* = 1.18, 95% CI [1.07, 1.31]), but WHZ was not (*OR* = 1.02, 95% CI [0.94, 1.12]).

**Table 3:** Odds of being on track for learning/cognition development with *z* scores of children, 36–59 months of age, in Bangladesh, Bhutan, Nepal, and Pakistan^a^

Variables	Model 1 (crude)^b^			Model 2^c^			Model 3^d^		
	*OR*	95% CI	*P*	*OR*	95% CI	*P*	*OR*	95% CI	*P*
HAZ									
Bangladesh	1.34	[1.23, 1.46]	<0.001	1.31	[1.20, 1.44]	<0.001	1.31	[1.18, 1.44]	<0.001
Bhutan	1.16	[0.93, 1.43]	0.19	1.15	[0.97, 1.37]	0.11	1.16	[0.98, 1.37]	0.08
Nepal	1.10	[0.98, 1.23]	0.11	1.05	[0.93, 1.18]	0.47	0.97	[0.85, 1.11]	0.64
Punjab, Pakistan	1.26	[1.17, 1.35]	<0.001	1.15	[1.06, 1.24]	<0.001	1.15	[1.07, 1.25]	<0.001
Sindh, Pakistan	1.43	[1.34, 1.53]	<0.001	1.23	[1.15, 1.32]	<0.001	1.22	[1.14, 1.31]	<0.001
All countries^e^ *I* ^2^				62.4%	*P* = 0.03		70.4%	*P* = 0.01	
All countries				1.19	[1.11, 1.27]	<0.001	1.17	[1.07, 1.27]	<0.001
WAZ									
Bangladesh	1.23	[1.11, 1.37]	<0.001	1.21	[1.09, 1.35]	0.001	1.21	[1.08, 1.35]	0.001
Bhutan	1.11	[0.92, 1.34]	0.27	1.1	[0.94, 1.34]	0.21	1.14	[0.95, 1.36]	0.16
Nepal	1.11	[0.96, 1.28]	0.16	1.01	[0.87, 1.16]	0.94	0.95	[0.82, 1.10]	0.48
Punjab, Pakistan	1.37	[1.17, 1.35]	<0.001	1.25	[1.14, 1.37]	<0.001	1.25	[1.15, 1.37]	<0.001
Sindh, Pakistan	1.56	[1.44, 1.70]	<0.001	1.34	[1.22, 1.46]	<0.001	1.33	[1.22, 1.46]	<0.001
All countries *I* ^2^				66.7%	*P* = 0.02		75.5%	*P* = 0.003	
All countries				1.19	[1.07, 1.33]	0.001	1.18	[1.07, 1.31]	0.002
WHZ									
Bangladesh	0.90	[0.81, 0.99]	0.04	0.91	[0.82, 1.01]	0.08	0.92	[0.83, 1.03]	0.15
Bhutan	0.98	[0.83, 1.16]	0.84	0.98	[0.84, 1.14]	0.78	0.99	[0.84, 1.16]	0.89
Nepal	1.03	[0.88, 1.19]	0.74	0.95	[0.83, 1.08]	0.40	0.95	[0.85, 1.08]	0.46
Punjab, Pakistan	1.19	[1.08, 1.32]	<0.001	1.14	[1.03, 1.26]	0.01	1.14	[1.03, 1.26]	0.01
Sindh, Pakistan	1.13	[1.03, 1.24]	0.01	1.09	[0.99, 1.19]	0.07	1.10	[1.01, 1.20]	0.04
All countries *I* ^2^				68.6%	*P* = 0.01		65.2%	*P* = 0.02	
All countries				1.02	[0.93, 1.11]	0.75	1.02	[0.94, 1.12]	0.59

a Learning/cognition development is considered to be on track if child can follow simple directions on how to do something correctly and/or when given something to do, and is able to do it independently.

b Model 1: Crude bivariate model.

c Model 2: Adjusted for location (rural vs. urban), household wealth quintile, household size, type of household head (male vs. female), improved drinking water source (yes/no), improved toilet facility (yes/no), maternal education, child age and sex, episodes of diarrhoea and cough in the past 2 weeks (yes/no), and study design effect.

d Model 3: In addition to all confounding variables used in Model 2, attendance to early childhood education, support for learning, availability of children's books, availability of playthings, and inadequate care were adjusted for in Model 3.

e *I* ^2^ is defined as the percentage of total variation across studies that is due to heterogeneity.

These associations of HAZ and WAZ with learning development were significant in Bangladesh (*OR* = 1.31, 95% CI [1.18, 1.44], and *OR* = 1.21, 95% CI [1.08, 1.35]), Punjab (*OR* = 1.15, 95% CI [1.07, 1.25], and *OR* = 1.25, 95% CI [1.15, 1.37]), and Sindh (*OR* = 1.22, 95% CI [1.14, 1.31], and *OR* = 1.33, 95% CI [1.22, 1.46]), but not in Bhutan and Nepal. WHZ was found to be associated with learning development in Punjab (*OR* = 1.14, 95% CI [1.03, 1.26]) and Sindh (*OR* = 1.10, 95% CI [1.02, 1.20]), but not in other countries.

#### 3.4.2 Social–emotional development

None of undernutrition indicators were associated with the social–emotional domain in the pooled sample (*OR* = 0.99, 95% CI [0.92, 1.07], for stunting; *OR* = 1.05, 95% CI [0.97, 1.14], for underweight; and *OR* = 1.07, 95% CI [0.86, 1.33], for wasting; Table 4). Unexpectedly, underweight and wasting were associated with higher odds of on‐track development in the social–emotional domain in Sindh, Pakistan (*OR* = 1.16, 95% CI [1.00, 1.34]) and Nepal (*OR* = 2.54, 95% CI [1.33, 4.85]), respectively. When adjusted for early childhood education and quality of care from Model 2 to Model 3, the association remained without attenuation in the pooled sample.

**Table 4:** Odds of being on track for social–emotional development among undernourished children relative to children of normal nutritional status, 36–59 months of age, in Bangladesh, Bhutan, Nepal, and Pakistan^a^

Variables	Model 1 (crude)^b^			Model 2^c^			Model 3^d^		
	*OR* e	95% CI	*P*	*OR*	95% CI	*P*	*OR*	95% CI	*P*
Stunting									
Bangladesh	1.01	[0.90, 1.13]	0.90	0.98	[0.87, 1.11]	0.73	0.98	[0.87, 1.11]	0.78
Bhutan	0.94	[0.74, 1.19]	0.60	0.94	[0.73, 1.19]	0.59	0.94	[0.74, 1.20]	0.62
Nepal	0.86	[0.63, 1.17]	0.34	0.95	[0.69, 1.29]	0.73	0.95	[0.69, 1.31]	0.76
Punjab, Pakistan	0.89	[0.78, 1.01]	0.07	0.93	[0.82, 1.07]	0.32	0.95	[0.83, 1.08]	0.43
Sindh, Pakistan	1.20	[1.01, 1.42]	0.04	1.11	[0.94, 1.32]	0.21	1.13	[0.95, 1.33]	0.17
All countries^f^ *I* ^2^				0.0%	*P* = 0.58		0.0%	*P* = 0.58	
All countries				0.98	[0.91, 1.06]	0.65	0.99	[0.92, 1.07]	0.83
Underweight									
Bangladesh	1.04	[0.91, 1.18]	0.59	1.04	[0.91, 1.19]	0.58	1.05	[0.91, 1.20]	0.53
Bhutan	0.96	[0.67, 1.37]	0.81	0.95	[0.66, 1.37]	0.79	0.93	[0.64, 1.35]	0.70
Nepal	1.05	[0.76, 1.46]	0.75	1.16	[0.83, 1.63]	0.38	1.20	[0.85, 1.68]	0.29
Punjab, Pakistan	0.92	[0.81, 1.04]	0.18	0.96	[0.84, 1.09]	0.49	0.97	[0.85, 1.10]	0.61
Sindh, Pakistan	1.21	[1.05, 1.41]	0.01	1.16	[1.00, 1.34]	0.05	1.16	[1.00, 1.34]	0.05
All countries *I* ^2^				9.1%	*P* = 0.36		9.8%	*P* = 0.35	
All countries				1.04	[0.96, 1.13]	0.31	1.05	[0.97, 1.14]	0.24
Wasting									
Bangladesh	1.14	[0.92, 1.42]	0.25	1.11	[0.89, 1.39]	0.36	1.10	[0.40, 0.88]	0.40
Bhutan	1.08	[0.58, 2.01]	0.80	1.10	[0.59, 2.05]	0.76	1.08	[0.58, 2.03]	0.80
Nepal	2.40	[1.25, 4.58]	0.01	2.50	[1.28, 4.87]	0.01	2.54	[1.33, 4.85]	0.01
Punjab, Pakistan	0.86	[0.73, 1.02]	0.08	0.87	[0.74, 1.03]	0.11	0.88	[0.74, 1.04]	0.13
Sindh, Pakistan	0.92	[0.74, 1.14]	0.44	0.93	[0.76, 1.15]	0.25	0.94	[0.76, 1.15]	0.53
All countries *I* ^2^				64.0%	*P* = 0.03		65.7%	*P* = 0.02	
All countries				1.05	[0.85, 1.30]	0.67	1.07	[0.86, 1.33]	0.57

a The child is considered on track in social–emotional development if two of the following are true: The child gets along well with other children; the child does not kick, bite, or hit other children; and the child does not get distracted easily.

b Model 1: Crude bivariate model.

c Model 2: Adjusted for location (rural vs. urban), household wealth quintile, household size, type of household head (male vs. female), improved drinking water source (yes/no), improved toilet facility (yes/no), maternal education, child age and sex, episodes of diarrhoea and cough in the past 2 weeks (yes/no), and study design effect.

d Model 3: In addition to all confounding variables used in Model 2, attendance to early childhood education, support for learning, availability of children's books, availability of playthings, and inadequate care were adjusted for in Model 3.

e For all odds ratio estimates, the reference groups are children who are not stunted (HAZ ≥ −2), underweight (WAZ ≥ −2), or wasted (WHZ ≥ −2).

f *I* ^2^ is defined as the percentage of total variation across studies that is due to heterogeneity.

None of the *z*‐score indicators were associated with social–emotional development in the pooled samples (all *p* > 0.05; Table 5). Except for a negative association between HAZ and social–emotional domain (*OR* = 0.95, 95% CI [0.90, 1.00]) in Sindh, Pakistan, no countries showed significant associations between *z*‐scores and social–emotional domain.

**Table 5:** Odds of being on track for social–emotional development among *z* scores of children, 36–59 months of age, in Bangladesh, Bhutan, Nepal, and Pakistan^a^

Variables	Model 1^b^			Model 2^c^			Model 3^d^		
	*OR*	95% CI	*P*	*OR*	95% CI	*P*	*OR*	95% CI	*P*
HAZ									
Bangladesh	0.99	[0.95, 1.04]	0.67	1.01	[0.96, 1.06]	0.81	1.00	[0.95, 1.06]	0.90
Bhutan	1.05	[0.96, 1.15]	0.32	1.06	[0.96, 1.16]	0.25	1.05	[0.96, 1.16]	0.26
Nepal	1.03	[0.92, 1.15]	0.61	1.00	[0.89, 1.12]	0.99	1.00	[0.89, 1.12]	0.98
Punjab, Pakistan	1.03	[0.99, 1.09]	0.17	1.01	[0.96, 1.07]	0.62	1.01	[0.96, 1.06]	0.79
Sindh, Pakistan	0.92	[0.88, 0.97]	0.002	0.95	[0.90, 1.00]	0.07	0.95	[0.90, 1.00]	0.04
All countries^e^ *I* ^2^				16.5%	*P* = 0.31		22.7%	*P* = 0.27	
All countries				1.00	[0.97, 1.03]	0.89	0.99	[0.963, 1.03]	0.72
WAZ									
Bangladesh	0.98	[0.92, 1.04]	0.55	1.00	[0.93, 1.07]	0.91	1.00	[0.93, 1.07]	0.99
Bhutan	1.05	[0.94, 1.17]	0.42	1.06	[0.94, 1.19]	0.34	1.07	[0.95, 1.20]	0.28
Nepal	1.02	[0.90, 1.16]	0.75	0.98	[0.86, 1.12]	0.81	0.98	[0.86, 1.12]	0.77
Punjab, Pakistan	1.04	[0.98, 1.10]	0.19	1.02	[0.96, 1.08]	0.59	1.01	[0.95, 1.07]	0.76
Sindh, Pakistan	0.94	[0.89, 0.99]	0.02	0.97	[0.91, 1.02]	0.24	0.96	[0.91, 1.02]	0.22
All countries *I* ^2^				0.0%	*P* = 0.63		0.0%	*P* = 0.60	
All countries				1.00	[0.96, 1.03]	0.84	1.00	[0.96, 1.03]	0.77
WHZ									
Bangladesh	0.96	[0.91, 1.02]	0.18	0.97	[0.91, 1.03]	0.28	0.98	[0.92, 1.04]	0.43
Bhutan	0.98	[0.90, 1.06]	0.57	0.99	[0.90, 1.07]	0.73	0.99	[0.91, 1.08]	0.85
Nepal	0.96	[0.87, 1.06]	0.45	0.96	[0.86, 1.06]	0.38	0.96	[0.86, 1.06]	0.37
Punjab, Pakistan	1.02	[0.96, 1.08]	0.55	1.01	[0.96, 1.08]	0.66	1.01	[0.95, 1.07]	0.74
Sindh, Pakistan	1.02	[0.95, 1.09]	0.60	1.01	[0.95, 1.08]	0.70	1.02	[0.95, 1.09]	0.63
All countries *I* ^2^				0.0%	*P* = 0.72		0.0%	*P* = 0.79	
All countries				0.99	[0.96, 1.02]	0.57	0.99	[0.96, 1.03]	0.71

a The child is considered on track in social–emotional development if two of the following are true: The child gets along well with other children; the child does not kick, bite, or hit other children; and the child does not get distracted easily.

b Model 1: Crude bivariate model.

c Model 2: Adjusted for location (rural vs. urban), household wealth quintile, household size, type of household head (male vs. female), improved drinking water source (yes/no), improved toilet facility (yes/no), maternal education, child age and sex, episodes of diarrhoea and cough in the past 2 weeks (yes/no), and study design effect.

d Model 3: In addition to all confounding variables used in Model 2, attendance to early childhood education, support for learning, availability of children's books, availability of playthings, and inadequate care were adjusted for in Model 3.

e *I* ^2^ is defined as the percentage of total variation across studies that is due to heterogeneity.

## 4 DISCUSSION

Our study provides a population‐level analysis of associations between nutrition indicators and learning/cognition and socioemotional developmental domains among children aged 36–59 months in South Asia, where childhood undernutrition and suboptimal development are prevalent. Although examined associations varied by developmental domain and country, across the region, stunted or underweight children were at greater risk of suboptimal learning/cognition development, as measured by the ECDI, than were children with adequate height and weight for age. The associations between *z* scores and learning developmental domain were consistent with those of corresponding undernutrition indicators.

Stunting, underweight, wasting, and *z* scores were not associated with social–emotional development in the pooled samples. In Bangladesh and the Punjab and Sindh provinces, stunting, underweight, HAZ, and WAZ were associated with learning developmental domain. In Sindh and Punjab, wasting and WHZ were also associated with learning development.

Our results align with literatures that continue to associate nutritional status with child development. A recent meta‐analysis of data from 68 cross‐sectional studies conducted in 29 LMICs reported associations between HAZ and suboptimal cognitive or motor development (Sudfeld et al., 2015). A study that examined child stunting and development indicators using MICS‐Round 4 data from 15 developing countries also reported negative associations between stunting/or severe stunting and learning/cognition development as well as a lack of consistent association with social–emotional development (Miller, Murray, Thomson, & Arbour, 2016). In addition to findings by Miller et al., our study used recent data from MICS Round 5 and included the associations between dichotomous (stunted, wasted, underweight) and continuous (*z* scores) indicators of undernutrition with child development indicators. Also, our study examined whether inclusion of child care and early education would change the association between child nutrition and developmental domains in South Asia.

Where examined, wasting shares an inconsistent relationship with child development indicators (Sudfeld, McCoy, Fink, et al., 2015). In our analysis, only Pakistan showed significant associations between wasting or WHZ and learning/cognition development, possibly due to higher burden of wasting (>11%) in the country than other countries (4.1~8.3%).

When early learning and better family care were introduced into our risk factor analyses, there were slight attenuations in otherwise strong negative associations between stunting and developmental indices, suggesting that early childhood care practices, as practised and reported in the surveyed home environments, may only partially ameliorate longer acting, negative effects of undernutrition on developmental capacity. The present findings support the implementation of interventions that may improve growth and reduce stunting in the first years of life to positive effects on early childhood development (Bhutta et al., 2013). Examples of such interventions include improved maternal nutrition to reduce the risk of maternal anaemia and small for gestational age or low weight birth, optimal breastfeeding and complementary feeding practices, micronutrient supplementation and food fortification, good hygiene and safe food handling practices, and timely detection and treatment of common childhood illnesses. The present findings also suggest that the integration of child stimulation, early learning, and better family care (Nguyen et al., 2017) with nutrition interventions is likely to yield the best results (Walker, Chang, Powell, & Grantham‐McGregor, 2005).

### 4.1 Strengths and limitations

Among limitations of this study is the cross‐sectional nature of the data, making it not possible to infer causality or mediation in the observed associations between child undernutrition and development indicators. It remains to be causally established that linear growth deceleration leading to stunting may adversely affect child development (Larson & Yousafzai, 2017; Perkins et al., 2017). Our analysis included four South Asian countries where MICS‐Round 4 or 5 was conducted. Data from other countries such as India, where 17.7 million children are estimated to have low cognitive and/or social emotional development (McCoy et al., 2016), were not included in this analysis, although regional comparability may permit a reasonable extension of inferences to similar population settings. There are likely to be unadjusted confounding variables, which could modify the observed associations between undernutrition and child development. Maternal nutrition and mental health, parental vital status, breastfeeding or complementary feeding history, environmental toxins, domestic violence, and disabilities are known examples of such confounding variables (Walker et al., 2011). However, these characteristics either have not been assessed or were assessed in only a few countries or in subsets of children in the MICS. The surprising lack of association, or even positive association (as seen in the Sindh Province) between the social–emotional maturity and undernutrition, raises questions about possible subjectivity and validity of the constructs of this domain, an issue meriting further studies. The ECDI tool used in the parent MICS is the first population‐level‐applicable tool to assess developmental domains at 3–4 years of age. Although the validity of the tool was assessed through many studies, it has some limitations. As previously mentioned, the literacy and physical domains in ECDI items were not age appropriate or detect only severe physical setbacks (McCoy et al., 2016). Subdomains of cognition (e.g., pattern recognition, memory, and languages), social skills, or culturally/context‐specific developmental milestones are not captured in the tool. As few items are assessed as yes/no for each developmental domain, interpersonal developmental differences or progress in developmental achievement at individual level are not reflected (McCoy et al., 2016).

## 5 CONCLUSIONS

In the South Asia region, stunting and underweight were associated with poorer learning developmental achievement among children 36–59 months old. On the contrary, none of nutrition indicators were associated with social–emotional development. Associations varied by domain and country. Our findings are consistent with emerging evidence that interventions that improve linear growth and reduce stunting may have a positive impact on development outcomes in infancy and early childhood. Integration of nutrition interventions and strategies to improve early stimulation and learning are likely to have synergistic positive impact on early childhood development and should be an investment priority in the South Asia region.

## CONFLICTS OF INTEREST

The authors declare that they have no conflict of interest. The contents expressed in the article are those of the authors and do not necessarily reflect the policies or views of the organizations with which they are affiliated.

## CONTRIBUTIONS

The authors' responsibilities were as follows: YK, RKC, VMA, and KW designed the current study. YK and RKC conducted data analysis for this study. YK wrote the manuscript. RKC, KW, and VMA reviewed the manuscript and substantially contributed to interpretation of the results. KW had primary responsibility for final content. All authors read and approved the final manuscript.

## Supporting information

Table S1. Z‐scores among children 36‐59 months of age in Bangladesh, Bhutan, Nepal, and Pakistan, Multiple Indicator Cluster Survey (MICS) 4 or MICS 5 (2010‐2014).
