# Supplementary material for: Association between stunting and early childhood development among children aged 36–59 months in South Asia
Source: Matern Child Nutr. 2018 Nov 29;14(Suppl 4):e12684. doi: 10.1111/mcn.12684 (PMC6588083; doi:10.1111/mcn.12684)
Supplement: Supplementary file 1 — Table S1. Z‐scores among children 36‐59 months of age in Bangladesh, Bhutan, Nepal, and Pakistan, Multiple Indicator Cluster Survey (MICS) 4 or MICS 5 (2010‐2014). [file MCN-14-e12684-s001.docx]

Supplemental Table 1. Z-scores among children 36-59 months of age in Bangladesh, Bhutan, Nepal, and Pakistan, Multiple Indicator Cluster Survey (MICS) 4 or MICS 5 (2010-2014).

|  | HAZ | WAZ | WHZ |
| --- | --- | --- | --- |
| Bangladesh | -1.90 (-1.94, -1.86) | -1.65 (-1.68, -1.61) | -0.74 (-0.77, -0.71) |
| Bhutan | -1.62 (-1.71, -1.53) | -1.02 (-1.09, -0.95) | -0.01 (-0.09, 0.07) |
| Nepal | -1.90 (-2.00, -1.80) | -1.52 (-1.60, -1.44) | -0.56 (-0.64, -0.48) |
| Punjab, Pakistan | -1.63 (-1.66, -1.59) | -1.58 (-1.61, -1.55) | -0.90 (-0.92, -0.87) |
| Sindh, Pakistan | -2.16 (-2.23, -2.09) | -1.84 (-1.90, -1.79) | -0.79 (-0.83, -0.74) |
